# Aspirin Administration Affects Neurochemical Characterization of Substance P-Like Immunoreactive (SP-LI) Nodose Ganglia Neurons Supplying the Porcine Stomach

**DOI:** 10.1155/2020/1049179

**Published:** 2020-06-12

**Authors:** Liliana Rytel, Jarosław Całka

**Affiliations:** ^1^Department of Internal Disease with Clinic, Faculty of Veterinary Medicine, University of Warmia and Mazury, Poland; ^2^Department of Clinical Physiology, Faculty of Veterinary Medicine, University of Warmia and Mazury, Poland

## Abstract

**Background:**

Acetylsalicylic acid (ASA) is a commonly used anti-inflammatory, antipyretic, and analgesic drug, which has many side effects on the gastric mucosal layer. Despite this, knowledge concerning the influence of ASA on neuronal cells supplying the stomach is very scanty.

**Methods:**

This investigation was performed on ten immature gilts of the Large White Polish race divided into two groups (five animals in each): a control group and animals which were treated with ASA. The retrograde neuronal tracer Fast Blue (FB) was injected into the prepyloric region of the stomach in all animals. ASA was then given orally to the experimental (ASA) group of gilts from the seventh day after FB injection to the 27th day of the experiment. After this period, all animals were euthanized. Immediately after euthanasia, nodose ganglia (NG) were collected and subjected to a standard double-labelling immunofluorescence technique using antibodies directed toward substance P (SP) and other selected neuronal factors, such as galanin (GAL), neuronal isoform of nitric oxide synthase (nNOS), vasoactive intestinal polypeptide (VIP), and calcitonin gene-related peptide (CGRP). *Key Results*. The obtained results show that SP-LI neurons located in NG supplying the porcine stomach were also immunoreactive to all the above-mentioned neuronal factors. Moreover, ASA administration caused an increase in the degree of colocalization of SP with other neuronal active substances, and the most visible changes concerned the number of neurons simultaneously immunoreactive to SP and CGRP. *Conclusions and Inferences*. These observations indicate that the population of SP-LI neurons supplying the stomach is not homogeneous and may undergo changes after ASA administration. These changes are probably connected with inflammatory processes and/or neuroprotective reactions although their exact mechanisms remain unknown.

## 1. Introduction

The innervation of the gastrointestinal (GI) tract consists of two main components. The first of them is the enteric nervous system (ENS) located in the wall of the digestive tract and built from the large number of neurons grouped in the enteric ganglia interconnected with a dense network of fibers and forming intramural ganglionated plexuses [1, 2]. The morphology and number of these plexuses depend on animal species and the segment of the digestive tract [3–6]. Two types of enteric ganglia can be identified in the stomach. The first of them is myenteric ganglia which, together with nerves connecting the particular ganglia, form a clearly visible myenteric plexus located between the longitudinal and circular muscle layers [7]. The other component of the gastric ENS is submucosal ganglia positioned near the lamina propria of the mucosal layer which, contrary to myenteric ganglia, do not form a plexus [7]. The second component of the gastrointestinal innervation is the extrinsic innervation. Neurons supplying the GI tract and controlling the activity of the ENS are localized (in accordance with their functions) in vagal ganglia, prevertebral sympathetic ganglia, and dorsal root ganglia [8–10].

It should be noted that both enteric neurons and cells participating in extrinsic intestinal innervation vary widely in terms of neurochemical characterization. To date, several dozen active substances have been noted in neuronal cells supplying the GI tract [11, 12]. One of the more important active substances occurring in neuronal cells supplying the gastrointestinal tract is substance P (SP) [13].

SP is an undecapeptide which, together with neurokinins A and B, as well as neuropeptides K and *γ*, belongs to the tachykinin neuropeptide family [14]. It was described for the first time in the 1930s [15], and since then, it has been observed in both intrinsic and extrinsic intestinal innervations in numerous mammal species, including humans [11, 16–18]. SP may act via three types of G protein-coupled NK receptors (NK1, NK2, and NK3), but the highest affinity is exhibited toward the NK1receptor. Previous studies showed that the exact functions of SP in the digestive tract depend on the intestinal fragment, animal species, and type of activated receptor [19, 20].

Multidirectional functions of SP in the regulation of the gastrointestinal activity have been described in previous studies. It is known that this substance takes part in the regulation of the smooth muscle contractility and the effects of this activity differ depending on the type of activated receptor. An SP-induced increase in the intestinal muscle contractility has been observed after excitation of the NK3 receptor, while the stimulation of the NK1 receptor causes relaxatory effects [21]. SP may also modulate the intestinal immunological system. This activity involves the activation of NK1 receptors located on lymphocyte and macrophage surfaces and an increase in the secretion of proinflammatory factors [22, 23]. Substance P is also involved in regulatory processes connected with intestinal secretory functions and mesenteric blood flow [24]. But the most important functions of the SP in the gastrointestinal innervation seem to be the participation in sensory and pain stimulus conduction [25]. Previous studies also reported that levels of substance P in neurons supplying the GI tract can undergo significant changes under various pathological factors, such as inflammatory processes, nerve damage, and other intestinal and extraintestinal diseases [11, 13].

It should be noted that, contrary to the enteric nervous system, which is located in the wall of the digestive tract where the distribution and functions of SP are relatively well established [26], knowledge concerning this peptide within the extrinsic gastrointestinal innervation is rather scarce. In particular, little is known about the neurochemical characterization of SP-positive neurons and changes in colocalization of SP with other active substances under pathological factors.

Thus, the present study was aimed at investigating the influence of acetylsalicylic acid (ASA) administration on the chemical coding of SP-like immunoreactive (SP-LI) neurons located in the nodose ganglia and supplying the porcine stomach. ASA (also known as aspirin) is widely used as anti-inflammatory, antipyretic, and analgesic medicine, which causes the inactivation of the cyclooxygenase (COX) enzyme [27]. Apart from therapeutic activity, aspirin also shows side effects which are particularly evident within the stomach, where taking this drug may result in the gastric mucosal injury [28]. It should be underlined that although the mechanisms of aspirin activity have been the subject of many studies [29], knowledge concerning its influence on extrinsic gastrointestinal innervation is extremely scarce [30]. On the other hand, it is relatively well established that aspirin may strongly affect the gastric mucosal layer, leading to its erosion and the breaking of the gastric mucosal barrier. These detrimental effects are based on the influence of ASA on the levels of adenosine triphosphate in mucosal cells, intensification of sodium transport, and changes in mucus composition, leading to injury of the gastric mucosal layer and the formation of gastric ulcers. Development of the local pathology evokes transduction of painful stimulation through the vagal sensory pathways that might affect the chemical plasticity of the nodose ganglion perikarya. Therefore, the objective of the current study was to analyze the effect of aspirin-induced stomach pathology on coexpression of galanin (GAL), neuronal isoform of nitric oxide synthase (nNOS), vasoactive intestinal polypeptide (VIP), and calcitonin gene-related peptide (CGRP) with substance P (SP) in nodose ganglion sensory perikarya [11].

## 2. Materials and Methods

### 2.1. Animals and Experimental Procedures

This investigation was performed on ten immature porcine of the Large White Polish breed (approximately 8 weeks old, about 20 kg b.w.), which were kept during the experiment in standard conditions suitable for the species and age of animals. All procedures during the study were performed in accordance with the instructions of the Local Ethical Committee in Olsztyn (Poland) (decision number 05/2010).

After a one-week adaptive period, the animals underwent general anesthesia with azaperone (Stresnil, Janssen Pharmaceutica N.V., Belgium; 4 mg/kg of body weight, i.m.) and sodium thiopental (Thiopental, Sandoz, Kundl-Rakusko, Austria; 10 mg/kg of b.w., i.v.) and gastroscopy examination (using the video endoscope Olympus GIF 145 with working length 1030 mm and diameter 9.8 mm) to evaluate the gastric mucosal layer. A median laparotomy was then conducted, and the prepyloric region of the anterior stomach wall (the diamond-shaped area of the approximate size 4 cm × 4 cm, located about 3 cm before the gastric pylorus) was injected with 50 *μ*l of a 5% aqueous solution of Fast Blue (FB, Dr. K. Illing GmbH & KG, Germany; ten injections, 1 *μ*l each) using a Hamilton syringe equipped with a 26-gauge needle. Great attention was paid to avoiding any contamination of the surrounding tissues with FB due to the hydrostatic leakage from the injection canal.

After the surgery, pigs were randomly divided into two groups of five animals in each: a control group (C group) and an ASA group, in which aspirin (Bayer; 100 mg/kg b.w.) was administered. Aspirin was given orally once a day, 1 h before the morning feeding from the seventh day after surgery to the 27^th^ day of the experiment. After this period (on the 28^th^ day of the experiment), the pigs were subjected to general anesthesia, euthanized with an overdose of sodium thiopental (Thiopental, Sandoz, Kundl, Austria; 20 mg/kg of body weight given intravenously), and perfused transcardially with 4% buffered paraformaldehyde (pH 7.4).

### 2.2. Tissue Collection

Right and left nodose ganglia (NG) were collected from all animals. Tissues were postfixed in 4% paraformaldehyde for 20 min, rinsed in M buffer solution (0.1 M, pH 7.4) for 72 hours, put into 18% sucrose solution, and stored at 4°C at least for three weeks. After this period, nodose ganglia were frozen at -22°C and cut using a microtome (Microm HM-525, Germany) into 12 *μ*m thick sections, which were evaluated under fluorescence Olympus BX51 for the presence of FB-positive neuronal cells.

### 2.3. Double Immunofluorescence Technique

Sections with FB-positive neurons were subjected to the routine double-labelling immunofluorescence method described previously by Rytel and Całka [11] with a mixture of two primary antibodies. One of them was an antibody directed toward substance P (rat, 1 : 150, AbD Serotec), and the second was an antibody directed toward one of the other neuronal factors, such as a calcitonin gene-related peptide (CGRP, rabbit, 1 : 4000, AbD Serotec), neuronal isoform of nitric oxide synthase (nNOS, used here as the marker of nitrergic neurons, rabbit, 1 : 4000, Chemicon), galanin (GAL, rabbit, 1 : 2000, Millipore), and vasoactive intestinal polypeptide (VIP, rabbit, 1 : 4000, BioGene). Complexes “primary antibody-appropriate antigen” were visualized by species-specific secondary antibodies conjugated with Alexa Fluor (Alexa Fluor 488 donkey anti-rat IgG and Alexa Fluor 546 donkey anti-rabbit IgG, Invitrogen, Carlsbad, CA, USA, working dilution 1 : 1000) (Table 1).

Standard control procedures of antibody specificity, including preabsorption, as well as “omission” and “replacement” tests, eliminated specific staining.

### 2.4. Evaluation of the Percentage of Neuronal Cells

Quantitative analysis was conducted using a fluorescence Olympus BX51 microscope with sets of filters for fluorochromes used in the present study. Microphotographs were taken using Cell-F image analysis software (Olympus, Tokyo, Japan). For evaluation of the degree of colocalization of SP with other neuronal factors within neurons supplying the stomach, at least 70 FB+/SP+ cell bodies in each nodose ganglion (left or right) were identified and examined for immunoreactivity to the particular substances investigated, and the number of FB+/SP+ neurons was treated as 100%. For example, unilateral identification in 85 animal FB+/SP+ cells was treated as 100% and was then performed (e.g., 22 of FB+/SP+/VIP+ neurons constituted 25.88%). Data obtained in animals of one group were pooled and presented as mean ± SEM.

The relatively low number of FB+/SP+ cells evaluated for the presence of the particular neuronal factors was caused by the fact that the population of SP-positive cells supplying the stomach located in the nodose ganglia is not numerous [31].

### 2.5. Statistical Analysis

Statistical analyses were performed using GraphPad Prism 5 software (GraphPad Software, USA) and an ANOVA test with Bonferroni's multiple comparison post hoc test. The differences were considered statistically significant at *P* ≤ 0.05.

### 2.6. Histopathological Examination

Moreover, routine histopathological staining with the application of the hematoxylin/eosin method was performed on fragments of the gastric wall from the prepyloric region to evaluate histopathological changes after ASA administration.

## 3. Results

During the present investigation, all neuronal active substances studied were observed in SP-positive neurons located in nodose ganglia and supplying the prepyloric region of the stomach, both under physiological conditions and after ASA administration.

**Figure 1:**
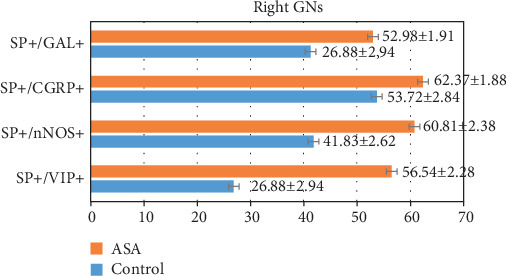
The number of SP-positive neurons and their neurochemical characterization in the right side of the nodose ganglia.

**Figure 2:**
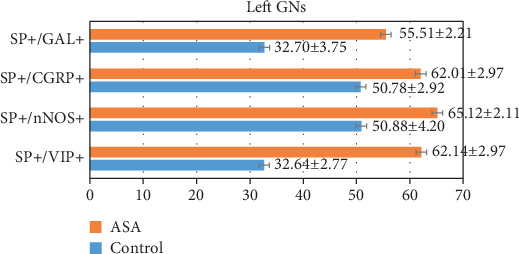
The number of SP-positive neurons and their neurochemical characterization in the left side of the nodose ganglia.

**Figure 3:**
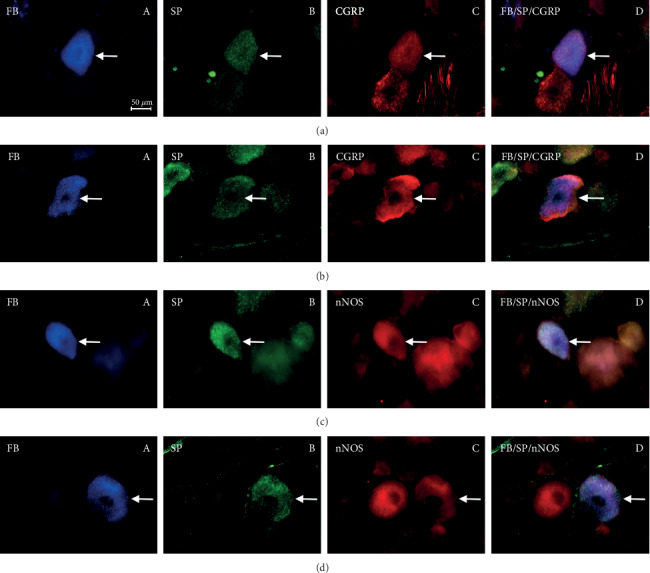
Fluorescence photographs showing perikarya (arrows) immunoreactive to the following: (a) FB (A), SP (B), and CGRP (C); (D) shows double labeling FB/SP/CGRP of the nodose ganglion neurons in the control group. (b) FB (A), SP (B), and CGRP (C); (D) shows double labeling FB/SP/CGRP of the nodose ganglion neurons in the ASA group. (c) FB (A), SP (B), and nNOS (C); (D) shows double labeling FB/SP/CGRP of the nodose ganglion neurons in the control group. (d) FB (A), SP (B), and nNOS (C); (D) shows double labeling FB/SP/CGRP of the nodose ganglion neurons in the ASA group.

**Figure 4:**
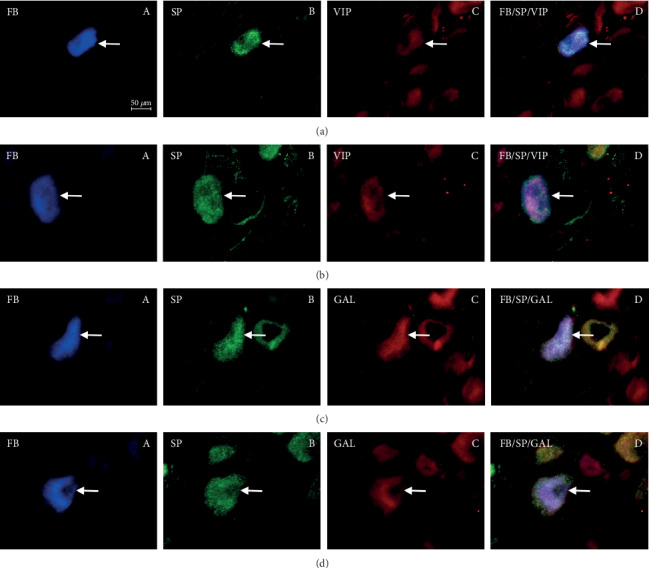
Fluorescence photographs showing perikarya (arrows) immunoreactive to the following: (a) FB (A), SP (B), and VIP (C); (D) shows double labeling FB/SP/CGRP of the nodose ganglion neurons in the control group. (b) FB (A), SP (B), and VIP (C); (D) shows double labeling FB/SP/CGRP of the nodose ganglion neurons in the ASA group. (c) FB (A), SP (B), and GAL (C); (D) shows double labeling FB/SP/CGRP of the nodose ganglion neurons in the control group. (d) FB (A), SP (B), and GAL (C); (D) shows double labeling FB/SP/CGRP of the nodose ganglion neurons in the ASA group.

In control animals, the degree of colocalization of SP with other substances depended on the type of substance and, to a lesser extent, on the side of nodose ganglion localization (Table 2) (Figures 1 and 2; Figures 3 and 4). The highest number of the investigated neurons contained CGRP, which was found within 53.72% ± 2.84% and 50.78% ± 2.92% of all FB+/SP+ neuronal cells in the right and left NG, respectively. Fewer neurons immunoreactive to SP and FB simultaneously show the presence of nNOS and/or GAL. In the right NG, nNOS was noted in 41.83% ± 2.62% of all FB+/SP+ neurons, and in the left, nNOS was noted in 50.88% ± 4.20%. In the case of GAL, these values amounted to 41.31% ± 3.55% and 32.70% ± 3.75%, respectively. The lowest percentage of SP-positive cells supplying the prepyloric region of the stomach showed immunoreactivity to VIP. FB+/SP+VIP+ neuronal cells accounted for 26.88% ± 2.94% and 32.64% ± 2.77% of all FB+/SP+ neurons in the right and left NG, respectively. Moreover, differences in the degree of colocalization of SP with other substances were noted between the right and left nodose ganglia. In the case of CGRP and/or GAL, the degree of colocalization was higher in the right NG, while a greater number of FB+/SP+ cells in the left NG simultaneously showed immunoreactivity to nNOS and/or VIP.

Aspirin administration changed the degree of colocalization of SP with all the investigated substances. Generally, these changes were manifested by the increase in the percentage of neurons immunoreactive to all substances studied in relation to all FB+/SP+ cells, but their intensity depended on the type of substance (Table 2) (Figures 1 and 2; Figures 3 and 4). The most visible changes concerned the degree of colocalization of SP and VIP. After ASA administration in the right NG, the percentage of FB+/SP+/VIP+ cells achieved 56.54% ± 2.28% of all cells immunoreactive to FB and SP (an increase of about 30 percentage points (pp) in comparison to control animals), and in the left NG, the percentage amounted to 62.14% ± 2.97% (an increase of above 30 pp). Less visible changes concerned the colocalization of SP with nNOS and/or CGRP. Under the influence of ASA, FB+/SP+/nNOS+ neuronal cells amounted to 60.81% ± 2.38% in the right NG (an increase of about 19 pp) and 65.12% ± 2.11% in the left NG (an increase of nearly 15 pp). For CGRP, these values achieved 62.37% ± 1.88% (an increase of about 9 pp) and 62.01% ± 2.97% (an increase of about 12 pp), respectively. The influence of aspirin on the degree of colocalization of SP with GAL appeared interesting. In particular, ASA administration caused only a slight increase in the percentage of FB+/SP+/GAL+ neurons in the right NG (from 41.31% ± 5.55% to 52.98% ± 1.91%, thus by about 11 pp) while within the left NG, the observed changes were relatively significant (an increase from 32.70% ± 3.75% to 55.51% ± 2.21%, thus by about 23 pp).

Moreover, microscopic inflammatory changes, such as hyperemia, edema, and lymphatic infiltration, were demonstrated during histopathological examination of the gastric mucosal layer in animals after aspirin administration. Long-term administration of ASA triggered hyperemia and numerous erosions and ulcerations in the mucosal surfaces of the stomach. Histopathological examination performed on the wall of the gastric prepyloric area collected from animals of the ASA group confirmed gastritis caused by the ASA treatment. Microscopic changes such as superficial erosions, hyperemia, infiltration of eosinophils, and proliferation of lymphocytein in the gastric mucosa were also observed.

## 4. Discussion

Previous studies have described how neurons supplying the prepyloric area of the porcine stomach are characterized by the presence of a wide range of active substances, including (among others) SP, CGRP, GAL, VIP, and nNOS. Long-term aspirin administration caused changes in the expression of all the studied substances [11]. The results of the present investigation confirm previous studies in which SP-positive neurons supplying the stomach were described in the nodose ganglia [11, 31]. Moreover, this study found that this neuronal population is not homogeneous and demonstrates a wide range of other neuronal substances, which colocalize with substance P. Since substances occurring in the same cells often play similar functions [11, 31], these observations may suggest that such a situation takes place in the case of SP and other substances studied during the present investigations. In control animals, the highest degree of colocalization concerned SP and CGRP. These two substances have been noted in the same neuronal cells within different parts of the central and peripheral nervous system, including (among others) the brain, dorsal root ganglia, enteric neurons, and prevertebral ganglia. This is not unusual, because CGRP (like SP) is known as one of the most important factors involved in sensory and/or pain stimulus conduction [32]. Moreover, both SP and CGRP in the digestive system may modulate the gastrointestinal secretory activity [33], blood flow in the wall of the digestive tract [34], and intestinal motility (although CGRP is not a typical factor regulating smooth muscle activity) [35].

Other substances observed during the present study in SP-positive neurons supplying the stomach may also show activities similar to actions of substance P. Namely, GAL takes part in the regulation of intestinal motility. The influence of GAL on the intestinal muscles may result in stimulatory or inhibitory effects depending on animal species and the intestinal fragment [36–38]. Similar situations have been described in the case of SP [39, 40]. Moreover, GAL (just like SP) is involved in regulatory processes connected with gastrointestinal secretory activity [41]. In turn, VIP and nitric oxide (in the present study, nNOS was used as a marker of nitrergic neurons) are known as the most important inhibitory factors within the gastrointestinal tract [42]. They influence the intestinal muscles causing relaxatory effects [42], reduce the secretory activity of the digestive tract [43], and regulate the mesenteric blood flow [44]. Moreover, the majority of substances studied in the present investigation are involved in immunological processes and show neuroprotective activity [45, 46]. It should be pointed out that the participation of SP and VIP in immunological processes shows an opposite character. Namely, SP is known as a potent proinflammatory factor [47], while VIP demonstrates anti-inflammatory activity [48]. The cooperation of the above-mentioned substances located in the same neurons in this respect remains unclear.

The obtained results show that aspirin administration may affect the neurochemical coding of neurons within the NG supplying the stomach, which is in agreement with previous studies [49]. Changes in neurochemical characterization of SP-positive neuronal cells may be connected with various mechanisms. The most commonly observed fluctuations are the result of the irritant effects of ASA on the gastric mucosal layer and/or inflammatory processes induced by this drug. Such activities of aspirin are relatively well known and have been described in previous studies [50]. Moreover, this inflammatory process has also been confirmed in the gastric mucosal layer during the present investigation. The possibility that the observed changes are the result of inflammation is supported by the fact that the majority of the studied substances take part in immunological processes and may affect the levels of pro- or anti-inflammatory factors [47, 48, 51, 52]. On the other hand, fluctuations in the chemical coding of SP-positive neurons may arise from the influence of inflammation on the conduction of sensory and pain stimuli. It is more likely that SP and CGRP are factors which are involved in sensory innervation [53] and nodose ganglia are typical sensory ganglia [54]. Of course, the other reasons for the observed changes cannot be excluded. They may be the result of the direct influence of ASA on sensory nerve endings in the gastric mucosal layer and manifestation of adaptive neuroprotective and/or reparative processes. This view is supported by two facts. Firstly, it is known that aspirin may affect the nervous system [49]. Secondly, the majority of substances studied in this investigation are involved in neuroprotective processes and neurogenesis after nerve damage during various pathological processes [45, 55]. Moreover, it is well established that the expression of factors involved in neuroprotective reactions usually increases under pathological factors [49, 55] and such changes were also noted during the present study. The reasons for the observed changes are also unclear. These changes could be the result of the inhibition of active substances being transported from the cell body to neuronal endings and synapses [56]. On the other hand, they may also be connected with fluctuations in neuropeptide synthesis, which may concern various stages of this process, such as transcription, translation, and posttranslational and/or metabolic disturbances [57].

In conclusion, the obtained results show that SP-positive neurons located in NG and supplying the stomach are differentiated according to the occurrence of other active substances, which likely function as comediators. Moreover, aspirin administration affects the neurochemical coding of these neurons. Changes observed under ASA action generally rely on the increase in the degree of colocalization of SP with other substances and are probably connected with inflammatory processes and/or neuroprotective activity. Nevertheless, due to the multidirectional activity of the neuronal substances studied and various pathological mechanisms connected with aspirin-induced changes, several aspects concerning the functioning of SP-LI neurons located in NG and innervating the stomach both under physiological conditions and during pathological processes remain unclear and require further studies.

## Figures and Tables

**Table 1:** Description of antibodies.

Antigen	Species of origin	Code	Dilution	Supplier
Primary antibodies				
GAL	Rabbit	AB2233	1 : 2000	Millipore
nNOS	Rabbit	AB5380	1 : 4000	Chemicon
VIP	Rabbit	VA 1285	1 : 4000	BioGene
SP	Rat	8450-0505	1 : 150	AbD Serotec
CGRP	Rabbit	AB5920	1 : 4000	AbD Serotec
Secondary antibodies				
Alexa Fluor 546	Donkey anti-rabbit	A10040	1 : 1000	Invitrogen
Alexa Fluor 488	Donkey anti-rat	A21208	1 : 1000	Invitrogen

**Table 2:** The number of SP-positive neurons and their neurochemical characterization in the right and left side of the nodose ganglia. The results were considered statistically significant at *P* ≤ 0.05.

Neuronal factor	Right GN		Left GN	
	Control	ASA	Control	ASA
SP+/VIP+	26.88 ± 2.94	56.54 ± 2.28^∗^	32.64 ± 2.77	62.14 ± 2.97^∗^
SP+/nNOS+	41.83 ± 2.62	60.81 ± 2.38^∗^	50.88 ± 4.20	65.12 ± 2.11^∗^
SP+/CGRP+	53.72 ± 2.84	62.37 ± 1.88^∗^	50.78 ± 2.92	62.01 ± 2.97^∗^
SP+/GAL+	41.31 ± 3.55	52.98 ± 1.91^∗^	32.70 ± 3.75	55.51 ± 2.21^∗^

## Data Availability

The data used to support the findings of this study are available from the corresponding author upon request.

## Abbreviations

SP: Substance P
NG: Nodose ganglia
FB: Fast Blue
CGRP: Calcitonin gene-related peptide
GAL: Galanin
nNOS: Neuronal isoform of nitric oxide synthase
VIP: Vasoactive intestinal polypeptide.

## Additional Points

*Key Points.* (1) Although the mechanisms of aspirin activity have been the subject of many studies, the knowledge concerning its influence on gastrointestinal innervation is extremely scanty. (2) Aspirin causes changes in all the investigated substances, and the most visible changes concerned the number of neurons immunoreactive to SP and VIP. (3) Aspirin shows effects which are particularly evident within the stomach, where taking this drug may result in the gastric mucosal injury.

## References

[1] Furness J. B., Callaghan B. P., Rivera L. R., Cho H. J. (2014). The enteric nervous system and gastrointestinal innervation: integrated local and central control. *Advances in Experimental Medicine and Biology*.

[2] Piasecki C. (1975). Observations on the submucous plexus and mucosal arteries of the dog's stomach and first part of the duodenum. *Journal of Anatomy*.

[3] Lolova I., Lolov N., Papasova M. (1980). Structure of the myenteric plexus in the sphincters of cat gastro-intestinal tract. III. Character and distribution of the dense-core vesicles in the axonal varicosities. *Acta Physiologica et Pharmacologica Bulgarica*.

[4] Phillips R. J., Powley T. L. (2012). Macrophages associated with the intrinsic and extrinsic autonomic innervation of the rat gastrointestinal tract. *Autonomic Neuroscience*.

[5] Kasacka I., Piotrowska Z., Car H., Janiuk I., Lebkowski W. (2012). Cocaine- and amphetamine-regulated transcript: identification and distribution in human gastrointestinal tract. *Journal of Biological Regulators and Homeostatic Agents*.

[6] Bódi N., Battonyai I., Talapka P., Fekete E., Bagyánszki M. (2009). Spatial pattern analysis of nitrergic neurons in the myenteric plexus of the duodenum of different mammalian species. *Acta Biologica Hungarica*.

[7] Furness J. B., Bornstein J. C., Smith T. K. (1990). The normal structure of gastrointestinal innervation. *Journal of Gastroenterology and Hepatology*.

[8] Hayakawa T., Kuwahara-Otani S., Maeda S., Tanaka K., Seki M. (2014). Brain-derived neurotrophic factor immunoreactive vagal sensory neurons innervating the gastrointestinal tract of the rat. *Journal of Chemical Neuroanatomy*.

[9] Ochoa-Cortes F., Guerrero-Alba R., Valdez-Morales E. E. (2014). Chronic stress mediators act synergistically on colonic nociceptive mouse dorsal root ganglia neurons to increase excitability. *Neurogastroenterology and Motility*.

[10] Miolan J. P., Niel J. P. (1996). The mammalian sympathetic prevertebral ganglia: integrative properties and role in the nervous control of digestive tract motility. *Journal of the Autonomic Nervous System*.

[11] Rytel L., Całka J. (2016). Acetylsalicylic acid-induced changes in the chemical coding of extrinsic sensory neurons supplying the prepyloric area of the porcine stomach. *Neuroscience Letters*.

[12] Palus K., Całka J. (2016). Neurochemical plasticity of the coeliac-superior mesenteric ganglion complex neurons projecting to the prepyloric area of the porcine stomach following hyperacidity. *Neural Plasticity*.

[13] Gonkowski S. (2013). Substance P as a neuronal factor in the enteric nervous system of the porcine descending colon in physiological conditions and during selected pathogenic processes. *BioFactors*.

[14] Mantyh P. W. (2002). Neurobiology of substance P and the NK1 receptor. *The Journal of Clinical Psychiatry*.

[15] Hökfelt T., Pernow B., Wahren J. (2001). Substance P: a pioneer amongst neuropeptides. *Journal of Internal Medicine*.

[16] Holzer P. (1985). Stimulation and inhibition of gastrointestinal propulsion induced by substance P and substance K in the rat. *British Journal of Pharmacology*.

[17] Gates T. S., Zimmerman R. P., Mantyh C. R. (1988). Substance P and substance K receptor binding sites in the human gastrointestinal tract: localization by autoradiography. *Peptides*.

[18] El-Salhy M., Spångéus A. (1998). Substance P in the gastrointestinal tract of non-obese diabetic mice. *Scandinavian Journal of Gastroenterology*.

[19] Karagiannides I., Pothoulakis C. (2009). Substance P, obesity, and gut inflammation. *Current Opinion in Endocrinology, Diabetes, and Obesity*.

[20] Barthó L., Holzer P. (1985). Search for a physiological role of substance P in gastrointestinal motility. *Neuroscience*.

[21] Goldhill J., Porquet M. F., Selve N. (1999). Antisecretory and relaxatory effects of tachykinin antagonists in the guinea-pig intestinal tract. *The Journal of Pharmacy and Pharmacology*.

[22] Reinke E. K., Johnson M. J., Ling C. (2006). Substance P receptor mediated maintenance of chronic inflammation in EAE. *Journal of Neuroimmunology*.

[23] Zhu J., Qu C., Lu X., Zhang S. (2014). Activation of microglia by histamine and substance P. *Cellular Physiology and Biochemistry*.

[24] Pawlik W. W., Gustaw P., Czarnobilski K., Sendur R., Konturek S. J. (1987). Effects of substance P on intestinal circulation and oxygen consumption. *Acta Physiologica Polonica*.

[25] Lai N. Y., Mills K., Chiu I. M. (2017). Sensory neuron regulation of gastrointestinal inflammation and bacterial host defence. *Journal of Internal Medicine*.

[26] Peng J., Li Y. J. (2010). The vanilloid receptor TRPV1: role in cardiovascular and gastrointestinal protection. *European Journal of Pharmacology*.

[27] Fiebich B. L., Lieb K., Kammerer N., Hüll M. (2004). Synergistic inhibitory effect of ascorbic acid and acetylsalicylic acid on prostaglandin E2 release in primary rat microglia. *Journal of Neurochemistry*.

[28] Tanaka M., Tanaka A., Suemaru K., Araki H. (2013). The assessment of risk for gastrointestinal injury with anticoagulant and antiplatelet drugs: the possible beneficial effect of eicosapentaenoic acid for the risk of gastrointestinal injury. *Biological & Pharmaceutical Bulletin*.

[29] Ng S. C., Chan F. K. L. (2010). NSAID-induced gastrointestinal and cardiovascular injury. *Current Opinion in Gastroenterology*.

[30] Tarnawski A. S., Ahluwalia A., Jones M. K. (2014). Increased susceptibility of aging gastric mucosa to injury: the mechanisms and clinical implications. *World Journal of Gastroenterology*.

[31] Zalecki M. (2014). Extrinsic primary afferent neurons projecting to the pylorus in the domestic pig--localization and neurochemical characteristics. *Journal of Molecular Neuroscience*.

[32] Matsunami H. (2012). CGRP*α*-expressing sensory neurons respond to stimuli that evoke sensations of pain and itch. *PLoS One*.

[33] Holzer P. (1998). Implications of tachykinins and calcitonin gene-related peptide in inflammatory bowel disease. *Digestion*.

[34] Uddman R., Edvinsson L., Ekblad E., Håkanson R., Sundler F. (1986). Calcitonin gene-related peptide (CGRP): perivascular distribution and vasodilatory effects. *Regulatory Peptides*.

[35] Demir I. E., Schäfer K. H., Tieftrunk E., Friess H., Ceyhan G. O. (2013). Neural plasticity in the gastrointestinal tract: chronic inflammation, neurotrophic signals, and hypersensitivity. *Acta Neuropathologica*.

[36] Korolkiewicz R. P., Konstański Z., Rekowski P. (2000). Sources of activator Ca2+ for galanin-induced contractions of rat gastric fundus, jejunum and colon. *Journal of Physiology and Pharmacology*.

[37] Zacharko-Siembida A., Valverde Piedra J. L., Szymańczyk S., Arciszewski M. B. (2013). Immunolocalization of NOS, VIP, galanin and SP in the small intestine of suckling pigs treated with red kidney bean (Phaseolus vulgaris) lectin. *Acta Histochemica*.

[38] Czujkowska A., Arciszewski M. B. (2016). Galanin is co-expressed with substance P, calbindin and corticotropin-releasing factor (CRF) in the enteric nervous system of the wild boar (Sus scrofa) small intestine. *Anatomia, Histologia, Embryologia*.

[39] Grider J. R. (1998). Regulation of excitatory neural input to longitudinal intestinal muscle by myenteric interneurons. *The American Journal of Physiology*.

[40] Nieber K., Oehme P., Milenov K. (1982). Different action of substance P on gastric and ileal smooth muscle. *Die Pharmazie*.

[41] Soldani G., Mengozzi G., Della Longa A., Intorre L., Martelli F., Brown D. R. (1988). An analysis of the effects of galanin on gastric acid secretion and plasma levels of gastrin in the dog. *European Journal of Pharmacology*.

[42] Van Geldre L. A., Lefebvre R. A. (2004). Interaction of NO and VIP in gastrointestinal smooth muscle relaxation. *Current Pharmaceutical Design*.

[43] Farthing M. J. (2006). Antisecretory drugs for diarrheal disease. *Digestive Diseases*.

[44] Maake C., Kaufmann C., Reinecke M. (2001). Ontogeny of neurohormonal peptides, serotonin, and nitric oxide synthase in the gastrointestinal neuroendocrine system of the axolotl (Ambystoma mexicanum): an immunohistochemical analysis. *General and Comparative Endocrinology*.

[45] Palus K., Całka J. (2016). Alterations of neurochemical expression of the coeliac-superior mesenteric ganglion complex (CSMG) neurons supplying the prepyloric region of the porcine stomach following partial stomach resection. *Journal of Chemical Neuroanatomy*.

[46] Barañano D. E., Snyder S. H. (2001). Neural roles for heme oxygenase: contrasts to nitric oxide synthase. *Proceedings of the National Academy of Sciences of the United States of America*.

[47] de Avila E. D., de Molon R. S., de Godoi Gonçalves D. A., Camparis C. M. (2014). Relationship between levels of neuropeptide substance P in periodontal disease and chronic pain: a literature review. *Journal of Investigative and Clinical Dentistry*.

[48] Kong P., Wu R., Liu X. (2016). The effects of anti-inflammatory drug treatment in gastric cancer prevention: an update of a meta-analysis. *Journal of Cancer*.

[49] Palus K., Całka J. (2015). The influence of prolonged acetylsalicylic acid supplementation-induced gastritis on the neurochemistry of the sympathetic neurons supplying prepyloric region of the porcine stomach. *PLoS One*.

[50] Thongbai T. (2013). The prevalence of gastroduodenal mucosal injuries in aspirin users. *Journal of the Medical Association of Thailand*.

[51] Edvinsson L. (2015). CGRP receptor antagonists and antibodies against CGRP and its receptor in migraine treatment. *British Journal of Clinical Pharmacology*.

[52] Lang R., Gundlach A. L., Holmes F. E. (2014). Physiology, signaling, and pharmacology of galanin peptides and receptors: three decades of emerging diversity. *Pharmacological Reviews*.

[53] Kestell G. R., Anderson R. L., Clarke J. N., Haberberger R. V., Gibbins I. L. (2015). Primary afferent neurons containing calcitonin gene-related peptide but not substance P in forepaw skin, dorsal root ganglia, and spinal cord of mice. *The Journal of Comparative Neurology*.

[54] Young R. L., Page A. J., Cooper N. J., Frisby C. L., Blackshaw L. A. (2010). Sensory and motor innervation of the crural diaphragm by the vagus nerves. *Gastroenterology*.

[55] Rytel L., Całka J. (2016). Neuropeptide profile changes in sensory neurones after partial prepyloric resection in pigs. *Annals of Anatomy*.

[56] Santafé M. M., Garcia N., Lanuza M. A., Tomàs J. (2007). Protein kinase C activity affects neurotransmitter release at polyinnervated neuromuscular synapses. *Journal of Neuroscience Research*.

[57] Onken H., Moffett S. B., Moffett D. F. (2004). The anterior stomach of larval mosquitoes (Aedes aegypti): effects of neuropeptides on transepithelial ion transport and muscular motility. *The Journal of Experimental Biology*.

